# Pyroptosis of MCF7 Cells Induced by the Secreted Factors of hUCMSCs

**DOI:** 10.1155/2018/5912194

**Published:** 2018-11-11

**Authors:** Yang Jiao, Hongbo Zhao, Guodong Chen, Xiongbo Sang, Luhan Yang, Zongliu Hou, Wei Si, Bingrong Zheng

**Affiliations:** ^1^School of Medicine, Yunnan University, Kunming, 650091 Yunnan, China; ^2^Yunnan Key Laboratory of Stem Cell and Regenerative Medicine, Institute of Molecular and Clinical medicine, Kunming Medical University, Kunming, 650500 Yunnan, China; ^3^Yan'an Hospital of Kunming City, Kunming, 650051 Yunnan, China; ^4^Yunnan Key Laboratory of Primate Biomedical Research, Institute of Primate Translational Medicine, Kunming University of Science and Technology, Kunming, 650500 Yunnan, China

## Abstract

Human umbilical cord mesenchymal stem cells (hUCMSCs) are superior to other sources of mesenchymal stem/stromal cells (MSCs), and they are used as a novel tool for cell-based cancer therapy. However, the mechanism underlying hUCMSC-induced cancer cell death is not clear. In the present study, we aimed to evaluate the effect of secreted factors of hUCMSCs on the breast cancer cell line MCF7 by exposing them to the conditioned medium (CM) of hUCMSCs. We evaluated the morphological changes, cell viability, cell cycle, apoptosis, DNA fragmentation, and interleukin-1*β* (IL-1*β*) secretion of CM-exposed MCF7 cells. The results showed that the secreted factors of hUCMSCs could cause MCF7 cell death by inducing pyroptosis. We also sequenced the total RNA, and the differentially expressed genes (DEGs) were subjected to the Kyoto Encyclopedia of Genes and Genomes (KEGG) pathway analysis. A total of 2597 (1822 upregulated and 775 downregulated) genes were identified and 14 pathways were significantly enriched. The results showed that the expression of the pyroptosis-related genes *NLRP1* and *CASP4* and the inflammation-related pathways changed significantly in MCF7 cells exposed to the CM. To the best of our knowledge, this study is the first to report that the secreted factors of hUCMSCs can cause MCF7 cell pyroptosis. Furthermore, it is the first to examine the global gene expression in MCF7 cells exposed to CM. These results will provide valuable information for further studies on the mechanism of MCF7 cell pyroptosis induced by the secreted factors of hUCMSCs. It will also help understand the effect of hUCMSCs on cell-based breast cancer therapy.

## 1. Introduction

Globally, breast cancer is the leading type of cancer among women, affecting approximately 2.1 million women [1] and resulting in 533,600 deaths in 2015 [2]. In China, there has been an increase in the incidence of breast cancer, and it is expected to account for 15% of new cancer cases [3]. Treatments for breast cancer include radiation therapy and surgery, followed by the administration of hormone-blocking agents, chemotherapy, and the use of monoclonal antibodies [4]. However, as breast cancers are classified by several grading systems, and as each of these can affect the prognosis and treatment response, a new effective treatment for breast cancer is necessary.

Pyroptosis is a type of programmed cell death and is distinct from the immunologically silent apoptotic cell death, which is caspase-1 dependent [5]. The activity of caspase-1 can result in the maturation of IL-1*β* and IL-18 and cleave gasdermin D to induce pore opening and pyroptosis [6]. Furthermore, inflammasomes are important for caspase-1 activity [7] and are composed of either AIM2-like receptor, tripartite motif-containing proteins, or the members of the nucleotide-binding domain, leucine-rich containing (NLR) family. The morphological changes during pyroptosis include plasma membrane rupture, water influx, cellular swelling, osmotic lysis, and proinflammatory cellular content release [8]. Furthermore, pyroptosis is different from apoptosis in terms of DNA cleavage, nuclear condensation, and nuclear integrity [8, 9].

Mesenchymal stem cells (MSCs) have received extensive attention as a new tool for cancer treatment. Human umbilical cord mesenchymal stem cells (hUCMSCs) are isolated from the human umbilical cord Wharton's jelly. The effects of hUCMSCs on cancer have been extensively studied. Han et al. [10] reported that hUCMSCs can induce apoptosis in PC-3 prostate cancer cells. Leng et al. [11] found that hUCMSCs can inhibit breast cancer progression by inducing tumor cell death and suppressing angiogenesis in mice. However, the mechanism underlying hUCMSC-induced cancer cell death is not clear. As secreted factors of hUCMCSs can inhibit cancer progression by inducing tumor cell death [12, 13], in the present study, we aimed to evaluate the effect of secreted factors of hUCMSCs on the breast cancer cell line MCF7, and we performed RNA-sequencing (RNA-Seq) to explore the genes and pathways involved in this process.

## 2. Materials and Methods

### 2.1. Cell Culture

The breast cancer cell line MCF7 used in the present study was obtained from the Kunming Cell Bank of the Chinese Academy of Sciences. It was maintained in Dulbecco's modified Eagle medium (DMEM) [containing 4.5 g/L glucose, L-glutamine, and 110 mg/L sodium pyruvate (Gibco by Thermo Fisher Scientific™, Suzhou, China)] supplemented with 10% fetal bovine serum (FBS, Gibco by Life Technologies™, Australia), 100 mg/L penicillin, and 100 mg/L streptomycin (Gibco by Life Technologies™, NY, USA) at 37°C with 5% CO_2_.

The hUCMSCs were obtained from the human umbilical cord Wharton's jelly by the tissue explant technique [14]. The umbilical cords were donated for study after informed patient consent and ethical approval by the Medical Ethics Committee of Yunnan University Medical School.

The hUCMSCs were cultured in Minimum Essential Medium Alpha Modification (*α*MEM, Hyclone by Thermo Scientific™, Beijing, China) supplemented with 10 ng/mL fibroblast growth factor basic protein (bFGF; Merck Millipore, Darmstadt, Germany), 100 mg/L penicillin, and 100 mg/L streptomycin at 37°C with 5% CO_2_. The identity of hUCMSCs was confirmed by the expression of mesenchymal cell surface markers CD105, CD90, CD44, and CK18 and by the nonexpression of CD45, HLA-DR, and CD31 (Additional file 1). The hUCMSCs were used before the eighth passage.

### 2.2. Exposure of MCF7 Cells to hUCMSC-Conditioned Medium

The hUCMSCs were cultured in plastic flasks (25 cm^2^, Corning, NY, USA). After 48 h (when the cells were approximately 90% confluent), the hUCMSCs cultured medium was collected and filter-sterilized using a 0.22 *μ*m Millex-GP Filter Unit (Millipore, Carrigtwohill, Tullagreen, Ireland). MCF7 cells at a density of 10^5^ cells/mL were seeded in 6-well plates (Corning, NY, USA) containing normal medium and cultured overnight. Subsequently, the medium was replaced with hUCMSC-conditioned medium (hUCMSC-CM). The hUCMSC-CM was prepared by gradually increasing the ratio of hUCMSC cultured medium to fresh medium (10% hUCMSC-cultured medium + 90% fresh medium, 20% hUCMSC-cultured medium + 80% fresh medium, 40% hUCMSC-cultured medium + 60% fresh medium, 60% hUCMSC-cultured medium + 40% fresh medium, 80% hUCMSC-cultured medium + 20% fresh medium, and 100% hUCMSC-cultured medium). The changes in MCF7 cell morphology were recorded and photographed on days 1, 2, 3, 4, and 5 using inverted phase contrast optics (LEICA DFC420C). The ratio of 80% hUCMSC-cultured medium and 20% fresh medium was chosen for further analysis as MCF7 cells exhibited obvious morphological changes and these changes were most synchronous in this medium.

### 2.3. MTS Analysis for Cell Viability

MCF7 cells were seeded at a seeding density of 5 × 10^3^ cells/well in 96-well plates (Corning, NY, USA) containing normal medium and cultured overnight. Subsequently, the medium was replaced with hUCMSC-CM (treatment) and fresh medium (control). The cell viability rate was evaluated using the CellTiter 96 Aqueous One Solution Cell Proliferation Assay kit (Promega, USA) on days 1, 2, 3, 4, and 5 after the replacement of medium according to the protocol of the manufacturer. Briefly, before the assay, 20 *μ*L of CellTiter 96 Aqueous One Solution Reagent was added into each well of the 96-well assay plate containing the samples in 100 *μ*L of culture medium, and the plate was then incubated at 37°C with 5% CO_2_. After 3 h, the absorbance was recorded at 490 nm.

### 2.4. Flow Cytometric Analysis for Cell Cycle

The control and MCF7 cells exposed to hUCMSC-CM for 48 h were collected after trypsin digestion without EDTA (Solarbio, Beijing, China) and fixed in ice-cold 70% ethanol overnight. The fixed cells were stained with 50 μg/mL propidium iodide (PI, CWBIAO, Beijing, China) in phosphate-buffered saline (PBS) containing 0.1% Triton X-100 and 50 *μ*g/mL RNase and then analyzed using the CyFlow Space (Partec) and FloMax 2.82 software.

### 2.5. Annexin V-FITC/PI Analysis

The percent of apoptotic cells was determined using the Annexin V-FITC/PI Apoptosis Detection kit (CWBIO, Beijing, China). Briefly, the control and MCF7 cells exposed to hUCMSC-CM for 48 or 72 h were collected after trypsin digestion without EDTA and then washed with cold PBS and resuspended in binding buffer at a concentration of 10^6^ cells/mL. The cells were incubated at 37°C with 5 *μ*L of Annexin V-fluorescein isothiocyanate (FITC) and 10 *μ*L of propidium iodide for 10 min and analyzed with the CyFlow Space (Partec) and FloMax 2.82 software.

### 2.6. Terminal Deoxynucleotidyl Transferase dUTP Nick-End Labeling (TUNEL) Assay

The fragmentation of DNA was analyzed using the DeadEnd fluorometric TUNEL assay kit (KeyGEN BioTECH, Nanjing, China) according to the protocol of the manufacturer. The control and MCF7 cells exposed to hUCMSC-CM for 72 h were fixed in 4% methanol-free formaldehyde solution in PBS for 30 min at 4°C and treated with proteinase K for 30 min at 37°C. Subsequently, the cells were treated with terminal deoxyribonucleotidyl transferase (TdT) containing biotin-11-dUTP for 1 h at 37°C in the dark and then treated with streptavidin-fluorescein for 30 min at 37°C in the dark. Finally, the cells were added to the medium containing 4′,6-diamidino-2-phenylindole and visualized under inverted phase contrast optics (LEICA DFC420C) after 10 min.

### 2.7. Quantification of Secreted IL-1*β* by the ELISA

The quantification of IL-1*β* secreted by MCF7 cells was carried out using the Human IL-1*β*/IL-1f2 Valukine™ ELISA Kit (NOVUS, Taiwan, China) according to the instructions of the manufacturer. The media from control MCF7 and MCF7 exposed to hUCMSC-CM were collected on days 0, 1, 2, 3, 4, and 5 and stored at −80°C for the enzyme-linked immunosorbent assay (ELISA).

### 2.8. RNA Sequencing

The control and MCF7 cells exposed to hUCMSC-CM (10^6^ cells/mL) were harvested (three biological replicates), and the total RNA was extracted using TRIzol™ reagent (Invitrogen, Carlsbad, USA). The RNA quality and quantity were determined using the RNA Nano 6000 Kit for the Agilent 2100 Bioanalyzer system, and the samples were stored at −80°C until further use. The total RNA (10 *μ*g) of each sample was used to construct the cDNA libraries. A total of six samples were sequenced using the BGISEQ-500 platform.

### 2.9. Bioinformatics Analysis

The raw reads were filtered using the internal software SOAPnuke to obtain clean reads [15]. The clean reads were then mapped with the reference genome using HISAT [16]. Gene expression was calculated as the number of uniquely mapped reads per kilobase of exon fragments per million mappable reads (FPKM) with RSEM [17]. Based on gene expression, the differentially expressed genes (DEGs) were analyzed using the DEGseq algorithms [18]. For the analysis, a *q* value of ≤0.001 and an absolute value of log_2_ ratio ≥ 1 were used to verify the significance of differences in gene expression. Based on the DEGs, the pathway functional analysis was performed using the clusterProfiler package [19]. The enrichment analysis was carried out by the hypergeometric test with a threshold value of 0.05 based on the KEGG database.

### 2.10. Validation of Gene Expression by the Quantitative Real-Time Polymerase Chain Reaction

MCF7 cells were cultured in hUCMSC-CM, and after 48 h, the total RNA from the cells was extracted using TRIzol™ reagent (Invitrogen, Carlsbad, USA). The quality and quantity of RNA were determined using the Nano-300 (Allsheng). All samples were then treated with the PrimeScript™ RT Reagent Kit with gDNA Eraser (Takara, China) to synthesize the first-stand cDNA. The primer sequences were designed using the PrimerQuest Tool (http://www.idtdna.com/primerquest/Home/Index), and the sequences are presented in Table 1. The quantitative real-time polymerase chain reaction (q-PCR) was performed using the BIO-RAD CFX96™ Real-Time System using FastStart Universal SYBR Green Master (Roche, Mannheim, Germany). Relative quantification was performed by the comparative Ct (2^-△△Ct^) method.

### 2.11. Statistical Analyses

The statistical analyses were performed using Microsoft excel 2007 and R 3.5.1, and graphs were drawn using the GraphPad Prism 5 software and R 3.5.1. *P* values *<*0.05 were considered statistically significant.

## 3. Results

### 3.1. Effects of hUCMSC-CM on MCF7 Morphology

We cultured MCF7 cells in different ratios of hUCMSC-cultured medium to fresh medium (Additional file 2). We found that even 10% hUCMSC-cultured medium and 90% fresh medium can cause MCF7 cells pore-induced plasma membrane invagination and then death. However, the number of dead cells was very small, no more than 50%. With an increase in the proportion of hUCMSC-cultured medium, the phenomenon of pore-induced plasma membrane invagination was more obvious and the number of dead cells increased. In the medium of 80% hUCMSC-cultured medium and 20% fresh medium, MCF7 cells showed obvious morphological changes and these changes were the most synchronous. Although MCF7 cells cultured in 100% hUCMSC-cultured medium have the same morphological changes and synchronization, the cells died too quickly. Therefore, we chose the ratio of 80% hUCMSC-cultured medium and 20% fresh medium for further analysis.

**Figure 1:**
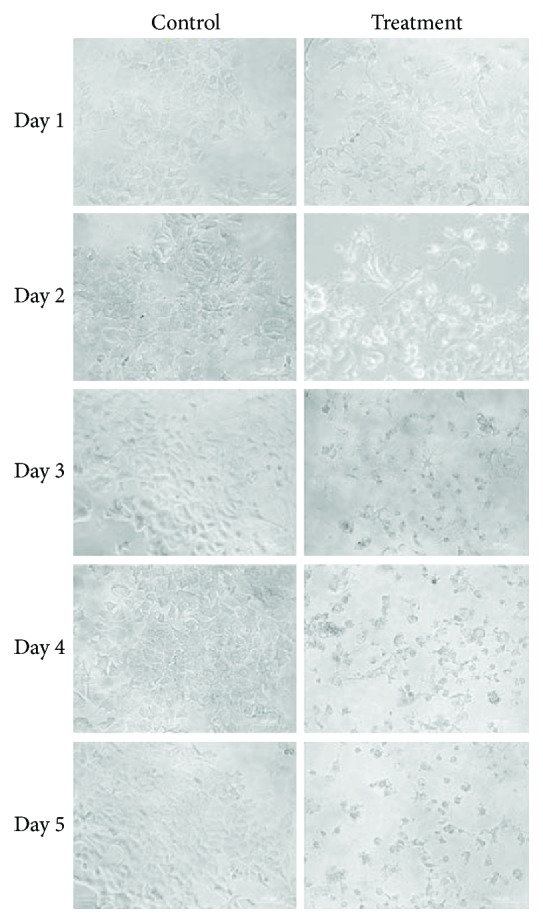
Comparative analysis of morphological changes in MCF7 cells exposed to hUCMSC-CM and control cells. Images were obtained on days 1, 2, 3, 4, and 5. Representative micrographs from at least three independent experiments are shown. All images were acquired at 20x magnification.

We observed a striking difference in the shape of MCF7 cells exposed to hUCMSC-CM and that of control MCF7 cells (Figure 1). The control MCF7 cells grew very well and continued to maintain their typical morphology, that is, the cell membrane was intact, the nucleus was not obvious, and the cells were polygonal and arranged orderly. However, MCF7 cells exposed to hUCMSC-CM gradually died, that is, the cells exhibited pore-induced plasma membrane invagination but still mainly intact at 24 h after the replacement of medium, and then the cells exhibited swelling at 48 h, and then the plasma membrane showed obvious ruptures, but the nucleus remained condense and the nuclear integrity was not compromised at 72 h.

### 3.2. Secreted Factors of hUCMSCs Caused MCF7 Cell Death but Did Not Change the Cell Cycle Significantly

**Figure 2:**
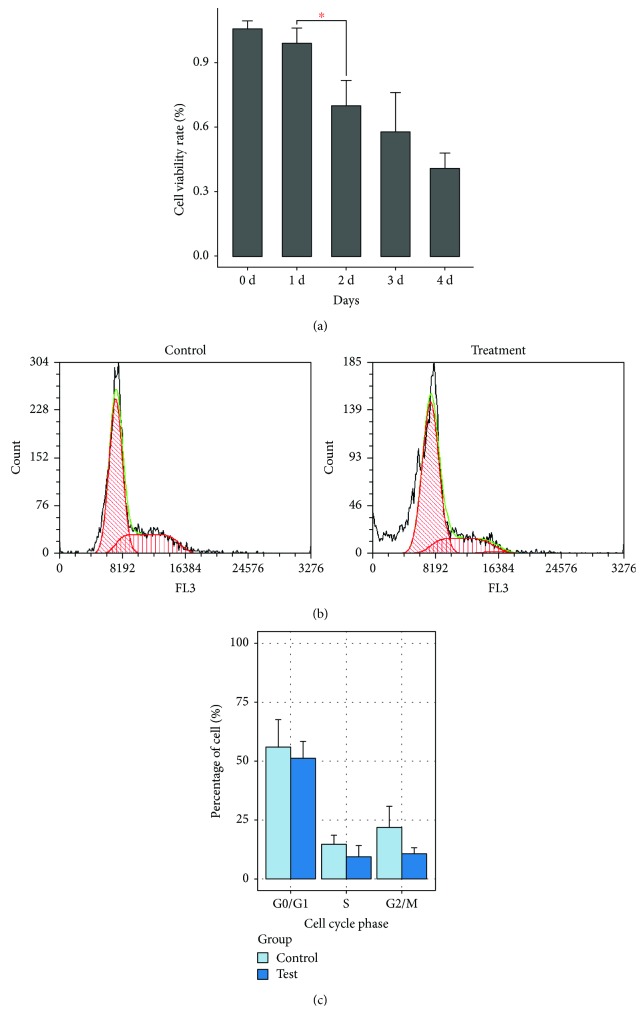
Cell viability and cell cycle analyses of MCF7 cells exposed to hUCMSC-CM (a) Effect of hUCMSC-CM on cell viability of MCF7 cells. *x*-axis represents days MCF7 cells exposed to hUCMSC-CM. *y*-axis represents cell viability rate. Cell viability rate = (the average OD of treatment group/the average OD of control group)^∗^100%. Results were measured using MTS proliferation method. Experiments were carried out in triplicate and repeated 3 times. ^∗^ *P* < 0.05. (b) Flow cytometric analysis for cell cycle of MCF7 cells exposed to hUCMSC-CM. Treatment cells were collected after 48 h, cultured in hUCMSC-CM, and stained with PI. (c) Quantitative analysis for cell cycle. Data were presented as mean ± SD of three independent experiments. hUCMSCs: human umbilical cord mesenchymal stem cells.

We determined the cell viability daily for 5 d after treatment. Compared with that of the control cells, the viability of MCF7 cells exposed to hUCMSC-CM decreased marginally on the first day and then decreased continually in the following days (Figure 2(a)). To verify whether the decrease in cell viability was caused by cell cycle arrest, the cell cycle of MCF7 cells at 48 h after the exposure to hUCMSC-CM was evaluated. The hUCMSC-CM-treated cells showed a marginal decrease in the G0/G1 (51.14 ± 7.27%), S (9.10 ± 5.01%), and G2/M phases (10.45 ± 2.80%) when compared with those of the control cells (55.96 ± 11.69%, 14.01 ± 4.58%, and 21.73 ± 8.90%, respectively) (Figure 2(b)); these cell cycle changes were not significant (Figure 2(c)). Therefore, the decrease in cell viability was due to an increase in the number of dead cells but not due to cell cycle arrest.

### 3.3. Secreted Factors of hUCMSCs Caused MCF7 Cells Death through the Pyroptosis Pathway

**Figure 3:**
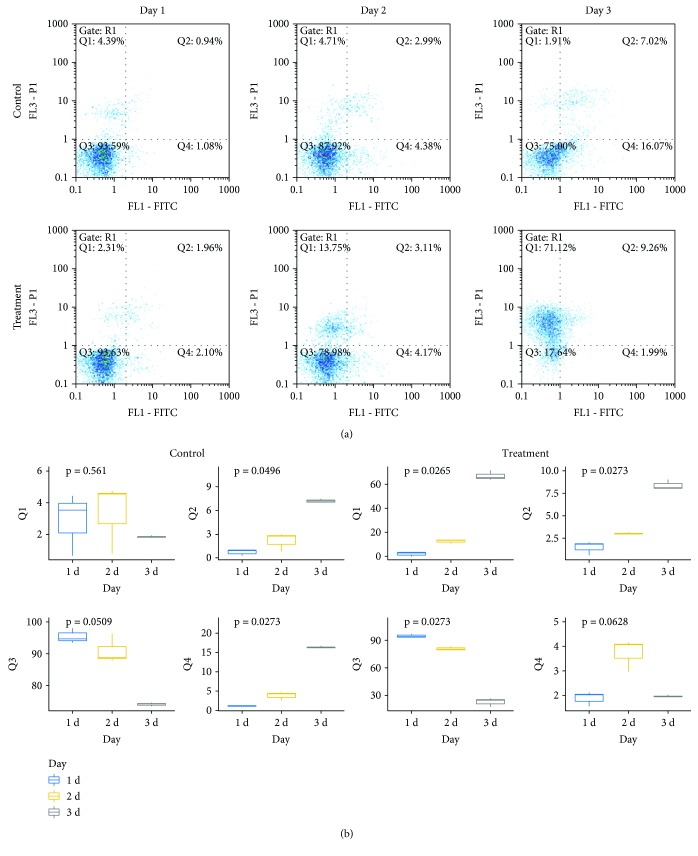
Flow cytometric analysis using Annexin V-FITC/PI. (a) Flow cytometry results of Annexin V-FITC/PI staining. Cells were collected on days 1, 2, and 3 after being exposed to hUCMSC-CM, respectively, and stained with 5 *μ*L each of Annexin V-FITC and 10 *μ*L PI. (b) Quantitative analysis for the Annexin V-FITC/PI results. Data were presented as mean ± SD of three independent experiments.

To elucidate how the secreted factors of hUCMSCs caused MCF7 cell death, we performed the Annexin V-FITC/PI analysis. The results showed that in the control cells, there was an increase in the number of early apoptotic and late apoptotic cells with time (*P* < 0.05). In the hUCMSC-CM-treated cells, the results showed that there was no obvious change in the FITC+/PI− quadrant, but there was an increase in the FITC+/PI+ quadrant, from 1.96% at 24 h to 9.26% at 72 h (*q* < 0.05). However, significant increases occurred in the FITC−/PI+ quadrant. It was only 2.31% at 24 h after the replacement of medium, which increased to 13.76% at 48 h and 71.12% at 72 h (Figure 3). Therefore, we conclude that the death of MCF7 cells exposed to hUCMSC-CM was not mainly mediated by apoptosis, and the increases occurred in the FITC−/PI+ quadrant due to the rupture of plasma membrane.

**Figure 4:**
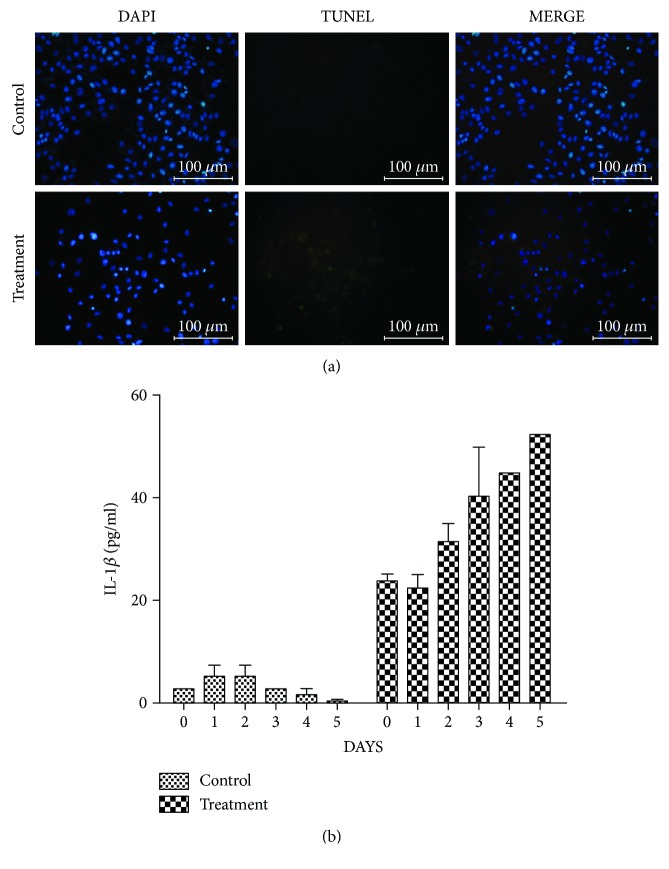
MCF7 cell assessment using TUNEL staining and secreted IL1*β* assay. (a) TUNEL assay for MCF7 cells at 72 h following exposure to hUCMSC-CM. (b) The concentration of secreted IL1*β* from MCF7 cells on days 0, 1, 2, 3, 4, and 5 following exposure to hUCMSC-CM. The experiment was repeated three times. *n* = 3.

As the morphological changes in MCF7 cells exposed to hUCMSC-CM were similar to those of pyroptotic cells described in the literature and the pyroptotic cells can be stained by PI [8], we further performed TUNEL staining and quantified secreted IL-1*β* to verify MCF7 cell death through the pyroptosis pathway. TUNEL staining labels DNA fragments. Like apoptotic cells, pyroptotic cells undergo DNA fragmentation and is stained positively with TUNEL [20]. The results showed that the control cells were negative for TUNEL staining, but MCF7 cells exposed to hUCMSC-CM were TUNEL positive (Figure 4(a)). The ELISA results showed that there was only a negligible amount of IL-1*β* in the medium of control cells (no more than 5 pg/mL), which gradually decreased. However, in the medium of hUCMSC-CM-treated cells, the initial concentration of IL-1*β* (25 pg/mL) was higher than that of the control. Subsequently, the concentration of IL-1*β* gradually increased, which doubled on day 5 (Figure 4(b)). These results suggest that the normal MCF7 cells do not secrete IL-1*β*, but MCF7 cells exposed to hUCMSC-CM secrete IL-1*β*, which is characteristic of pyroptotic cells [21]. Therefore, we conclude that the secreted factors of hUCMSCs caused MCF7 cell death through the pyroptosis pathway.

### 3.4. DEGs Were Analyzed and 14 Significant Pathways Were Detected according to RNA-Seq Data

The RNA samples from MCF7 cells, both control and treated cells (the data were obtained from three biological replications), were sequenced using the BGISEQ-500 platform. On an average, approximately 23.99 Mb reads per sample were generated and 18,174 genes were detected. Furthermore, 23.93 Mb clean reads were obtained after filtering the data. The average mapping ratio of clean reads with reference human genome was 94.63%, and the uniformity of the mapping results for each sample suggested that the samples were comparable.

**Figure 5:**
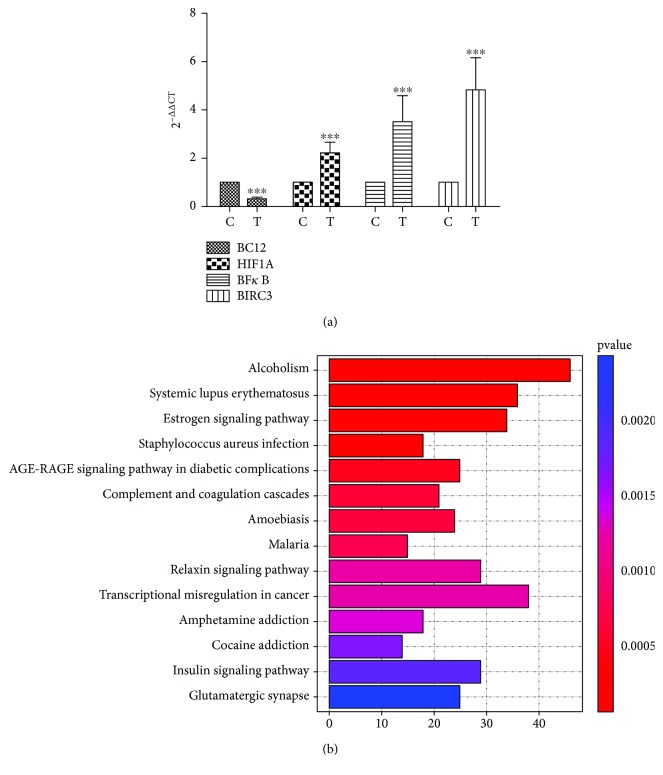
Gene expression and KEGG pathway analysis. (a) Validation of selected genes from DEGs by q-PCR. Data are presented as Ct (2^-△△Ct^) relative to control. Data are presented as mean ± S.D. *n* = 3. ^∗^ *P* < 0.05, ^∗∗^ *P* < 0.01, and ^∗∗∗^ *P* < 0.001. C: control group; T: treatment group. (b) The significant KEGG pathways (*P* < 0.001). *x*-axis represents number of genes. *y*-axis represents pathways.

In order to find DEGs, gene expression of each sample was calculated as FPKM with RSEM and the DEGs were identified using the DEGseq algorithms. A total of 1822 up- and 775 downregulated genes were identified (|log_2_ (fold change)| > 1, *q* value ≤0.001). The genes *NLRP1*, *CASP4*, *TLR2*, *TLR6*, *NFκB*, *HIF1A*, and *BIRC3* were significantly upregulated, and the *Bcl2* gene was significantly downregulated (Table 2). To validate gene expression, we randomly chose the upregulated genes *HIF-1A*, *NF-κB*, and *BIRC3* and the downregulated gene *Bcl2* to do the q-PCR. The specificity of q-PCR primers was confirmed by regular PCR (Additional file 3). Hypoxia-inducible factor1-alpha (HIF-1A) is a master transcriptional regulator of cellular and developmental response to hypoxia [22]. Nuclear factor kappa-light-chain-enhancer of activated B cells (NF-*κ*B) is a protein complex that controls transcription of DNA, cytokine production, and cell survival. It is involved in cellular responses to stimuli and plays a key role in regulating the immune response to infection [23]. Baculoviral IAP repeat-containing protein 3 (BIRC3) is a member of the inhibitor of apoptosis family that inhibits apoptosis by interfering with the activation of caspases [24]. The *Bcl2* gene is a member of the *Bcl2* family of regulator proteins that regulate cell death, by either inducing (proapoptotic) or inhibiting (antiapoptotic) apoptosis [25]. The q-PCR results showed that differential gene expression was consistent with the RNA-Seq results (Figure 5(a)).

To identify the associated biological functions, the DEGs were subjected to the KEGG pathway analysis. The pathway functional enrichment results (Figure 5(b)) showed that there are 14 significant pathways (*P* < 0.05), including alcoholism, systemic lupus erythematosus, estrogen signaling pathway, *Staphylococcus aureus* infection, AGE-RAGE signaling pathway in diabetic complications, complement and coagulation cascades, amoebiasis, malaria, relaxin signaling pathway, transcriptional misregulation in cancer, amphetamine addiction, cocaine addiction, insulin signaling pathway, and glutamatergic synapse.

## 4. Discussion

The MSCs are multipotent stromal cells, which can be obtained from the bone marrow [26], adipose tissue [27], umbilical cord tissue, and umbilical cord blood [28]. In terms of therapeutic applications, the hUCMSCs have some beneficial properties when compared with those of other MSCs. These properties include large-cell number at harvest, low immunogenicity, propagated without feeder cells, stored after birth without significant risks to the donor, and absence of ethical dispute. In vivo, the MSCs exhibit oriented migration to the site of tumor, and they inhibit tumor growth and metastasis [11, 29, 30]. Therefore, the MSCs have been considered potential biological vehicles for tumor-targeted delivery of therapeutic genes [31–33]. In vitro, the effects of MSCs on cancer cells have been studied either by coculturing cancer cells with MSCs or by culturing cancer cells in CM, prepared using fresh medium and medium in which MSCs were cultured for several days. In the present study, we cultured MCF7 cells in hUCMSC-CM to study their effects. To the best of our knowledge, we report for the first time that the secreted factors of hUCMSCs can induce MCF7 cell pyroptosis. We also examined gene expression in MCF7 cells exposed to CM by RNA-Seq.

The changes in the morphology of MCF7 cells exposed to hUCMSC-CM revealed the characteristics of pyroptosis, that is, they had a permeable membrane which was initially intact, which was followed by cell swelling, rapid expansion, and finally rupture [34]. During this process, the nucleus of MCF7 cells exposed to hUCMSC-CM condensed and the DNA fragmented. As the plasma membrane ruptured, the cells were detected by PI staining [8]. As apoptotic cells, the pyroptosis cells were TUNEL positive [20]. And also, we found an obvious increase in the amount of IL-1*β* in the medium of MCF7 cells exposed to hUCMSC-CM. To summarize, we considered that MCF7 cells exposed to hUCMSC-CM were undergoing pyroptosis [21]. In the present study, MCF7 cells exposed to hUCMSC-CM showed swelling at 48 h and ruptured at 72 h. Therefore, the viability of MCF7 cells decreased 48 h after the replacement of medium. The treated cells showed no obvious changes in cell cycle. This indicates that hUCMSC-CM cannot arrest the cell cycle of MCF7 cells.

The RNA-Seq results revealed that 1822 and 775 genes were up- and downregulated in MCF7 cells exposed to hUCMSC-CM, respectively, compared with those of the control MCF7 cells. According to the KEGG analysis, we identified 14 significant pathways. We further classified these pathways according to the KEGG PATHWAY Database (http://www.genome.jp/kegg/pathway.html). We found that they are mainly related to the immune system, endocrine system, nervous system, and the diseases associated with these systems. The results showed that certain substances in hUCMSC-CM could cause inflammatory reaction and immune response in MCF7 cells. Whereas, some substances can cause endocrine system disorder, which is mainly induced by estrogen, relaxin, or glycometabolism, and some other substances can cause behavioral responses in MCF7 cells on exposure to addictive substances.

Interestingly, we found that the *NLRP1* gene was significantly upregulated. Nucleotide-binding, leucine-rich repeat pyrin domain-containing protein 1 (*NLRP1*) is a member of the NOD-like receptor (NLR) family and plays an important role in cell pyroptosis. As an important natural immune molecule, NLRP1 can identify pathogen-associated molecular patterns and risk molecular models as a pattern recognition receptor [35]. In humans, *NLRP1* has the ability to activate *caspase-1* by interacting directly with procaspase-1 or by recruiting adaptor protein ASC and then recruiting procaspase-1 to form a complex known as inflammasome [36]. Activated *caspase-1* can help in the maturation of pro-IL-1*β* and pro-IL-18 [37], causing inflammation to promote pyroptosis [38]. According to the RNA-Seq results, *caspase-1* was neither up- nor downregulated. This indicates that *caspase-1* may affect pyroptosis by activating procaspase-1 and not by increasing the transcriptional product. However, *caspase-4*, a member of a cysteine protease family called caspases, was significantly upregulated. In humans, *caspase-4* functions as the receptor of lipopolysaccharide (LPS) [39], which is found in the outer membrane of gram-negative bacteria and can induce pyroptosis [6]. When LPS binds to caspase-4, caspase-4 undergoes oligomerization, leading to caspase-1-induced maturation of IL-1*β* and IL-18, thereby mediating pyroptosis [40]. The significant upregulation of *caspase-4* shows that *caspase-4* might play an important role in the inflammation and pyroptosis of MCF7 cells induced by hUCMSC-CM. From the KEGG analysis, we found that systemic lupus erythematosus (SLE) is the second significant pathway. It is an autoimmune disease and is confirmed to be associated with the NLR family members, such as *NLRP1* and *NLRP3*. We also found that the assembly of inflammasomes is induced by NLR [41, 42]. Excessive inflammasome activation can promote excessive release of IL-1*β* and IL-18, causing autoinflammatory disorders and the subsequent development of autoimmune diseases, such as SLE [42]. Therefore, the enrichment of this pathway confirms that when MCF7 cells are cultured in hUCMSC-CM, they are subjected to inflammation.

TLR2, the other receptor related to initial immunization, was significantly upregulated. TLR2 is a member of the Toll-like receptor (TLR) family. It is expressed on the surface of certain cells, recognizes pathogen-associated molecular patterns and mediates the production of cytokines necessary for the development of effective immunity [43]. TLR2 can bind to a wide range of exogenous ligands, including lipoproteins, lipoteichoic acid, peptidoglycan, zymosan, lipoarabinomannan, glycosylphosphatidylinositol, phenol-soluble modulin, and glycolipids [44], thereby mediating host immune response against gram-positive bacteria [45]. This function of TLR2 is unique in its ability to form TLR2/TLR1 or TLR2/TLR6 heterodimer [46]. The TLR2-ligand interactions activate MyD88 signaling, which then activates proinflammatory transcription factors, such as NF-*κ*B and the PI3K/AKT pathway [47]. NF-*κ*B translocates into the nucleus to regulate the production of inflammatory cytokines, such as IL-1*β*, IL-6, IL-8, and IL-12 [44]. The stimulation by TLR2-ligands alone without a second signal can promote IL-1*β* release but cannot induce cell death [48]. From the results of RNA-Seq, we also found that the expression of TLR6 and NF-*κ*B was significantly upregulated. This indicates that the inflammation caused by TLR2 is not the major pathway that induces MCF7 cell pyroptosis but might play an important role in response to the stimulation of secreted factors of hUCMSCs and assist in MCF7 cell death. From the KEGG analysis, *Staphylococcus aureus* infection was found to be the fourth significant pathway. *Staphylococcus aureus* is a gram-positive bacterium and can be recognized by *TLR2*, indicating that *TLR2* is indeed an important gene in the death of MCF7 cells exposed to hUCMSC-CM.

Hypoxia-inducible factor1-alpha (HIF-1A) is a master transcriptional regulator of cellular and developmental response to hypoxia [22]. The activity of HIF-1A is directly regulated by cellular oxygen levels [49], and under hypoxic condition, *HIF-1A* transcription is often significantly upregulated [50, 51]. According to the RNA-Seq result, the expression of *HIF-1A* was upregulated. This indicates that when MCF7 cells are cultured in hUCMSC-CM, a hypoxic environment is created within the cells. Jorgensen et al. [34] found mitochondria with collapsed cristae in pyroptotic cells. Mitochondria are the major site of ROS production. They have a four-layer structure, and ROS generation mainly occurs at the electron transport chain (ETC) located on the inner mitochondrial membrane, which folds inward to form a ridge-like shape and called cristae, during the process of oxidative phosphorylation (OXPHOS) [52]. Therefore, the collapsed cristae could lead to reduction in ROS production [53], thereby causing hypoxia in cells. In fact, hypoxia causes pyroptosis. Wu et al. found hypoxia-reoxygenation injury can induce pyroptosis in HK2 cells [54]. In addition, Qiu et al. demonstrated that stimulation of H9C2 cardiomyocytes with high glucose and hypoxia/reoxygenation increased NLRP3 inflammasome activation and pyroptosis [55]. Furthermore, as autophagic suppression of ROS can limit ROS-modulated caspase-1 activation to inhibit inflammasome activity [56], hypoxia also might be a negative feedback mechanism in response to high inflammation within MCF7 cells. Recent studies have demonstrated that the expression of *HIF-1A* is regulated by the NF-*κ*B [57], and *HIF-1A* restricts NF-*κ*B transcriptional activity, thereby preventing inflammation and controlling the innate immune response [58, 59]. This indicates that the upregulation of *HIF-1A* in MCF7 cells exposed to hUCMSC-CM might be a negative feedback mechanism activated in response to high inflammation inside MCF7 cells.

There are a few limitations to this study. We selected only one breast cancer cell line to validate the expression of key genes. The use of MCF7 cells started in 1973, and they are widely used due to their exquisite hormone sensitivity [60]. However, the breast cancer cell lines have different subtypes, in which the expression of key genes should be validated. Furthermore, we only studied gene expression in pyroptotic MCF7 cells by RNA-Seq. Further analysis should be carried out for a deeper understanding.

## 5. Conclusions

The present study demonstrates that the secreted factors of hUCMSCs can promote inflammatory response and pyroptosis in MCF7 cells. Therefore, the inflammation- and hypoxia-related genes and pathways play an important role in MCF7 cell pyroptosis. Although further studies are necessary to characterize the interaction of these genes and pathways, our data provides valuable data for further studies in this direction.

## Figures and Tables

**Table 1:** Primer sequences for quantitative real-time polymerase chain reaction.

Primers	NCBI ID	Primer sequences
Bcl2	NM_000633.2	S: 5′ TTTCTCATGGCTGTCCTTCAGGGT 3′ A: 5′ AGGTCTGGCTTCATACCACAGGTT 3′
HIF1A	NM_001530.3	S: 5′ GTCTGCAACATGGAAGGTATTG 3′ A: 5′ GCAGGTCATAGGTGGTTTCT 3′
NF-*κ*B	NM_002502.5	S: 5′ GAAGATTGAGCGGCCTGTAA 3′ A: 5′ TGTCTTCCACCAGAGGGTAATA 3′
BIRC3	NM_001165.4	S: 5′ CAAGCCAGTTACCCTCATCTAC 3′ A: 5′ CTGAATGGTCTTCTCCAGGTTC 3′

**Table 2:** List of differentially expressed genes in MCF7 cells exposed to hUCMSC-CM.

Symbol	Gene ID	Length (bp)	Control expression	Treatment expression	log2 ratio (treatment/control)	*q* value
NLRP1	22,861	5491	20	102	2.35	8.40E-15
CASP4	837	1319	75	253	1.75	7.10E-24
TLR2	7097	8147	61.43	142.99	1.22	5.10E-09
TLR6	10,333	6683	60.31	124.52	1.04	1.12E-06
NF*κ*B	4791	3067	6298	14,367	1.19	0
HIF1A	3091	4070	5694	19,529	1.78	0
BIRC3	330	5174	227.6	2297.57	3.33	0
Bcl2	596	5191	1546.17	383	−2.02	1.35E-164

## Data Availability

The datasets generated and analyzed in the current study are included in within the article. The raw data of the RNA-Seq analysis will be provided by the corresponding author on request.

## Supplementary Materials

Supplementary 1 Additional file 1: flow cytometry analysis of mesenchymal stem cells surface markers. The flow cytometry analysis shows expression of CD105, CD90, CD44, and CK18 and nonexpression of CD 45, HLA-DR, and CD31 on the hUCMSC surface.

Supplementary 2 Additional file 2: confirm the phenotype using gradually increased ratio of hUCMSC-medium to fresh medium. Control: 100% fresh medium, treatment 1: 10% hUCMSC-medium + 90% fresh medium, treatment 2: 20% hUCMSC-medium + 80% fresh medium, treatment 3: 40% hUCMSC-medium + 60% fresh medium, treatment 4: 60% hUCMSC-medium + 40% fresh medium, treatment 5: 80% hUCMSC-medium + 20% fresh medium, treatment 6: 100% hUCMSC-medium.

Supplementary 3 Additional file 3: confirm the specificity of q-PCR primers using regular PCR. DNA marker: DL2000.
