# Supplementary material for: Pyroptosis of MCF7 Cells Induced by the Secreted Factors of hUCMSCs
Source: Stem Cells Int. 2018 Nov 11;2018:5912194. doi: 10.1155/2018/5912194 (PMC6252231; doi:10.1155/2018/5912194)
Supplement: Supplementary 1 — Additional file 1: flow cytometry analysis of mesenchymal stem cells surface markers. The flow cytometry analysis shows expression of CD105, CD90, CD44, and CK18 and nonexpression of CD 45, HLA-DR, and CD31 on the hUCMSC surface. [file 5912194.f1.docx]

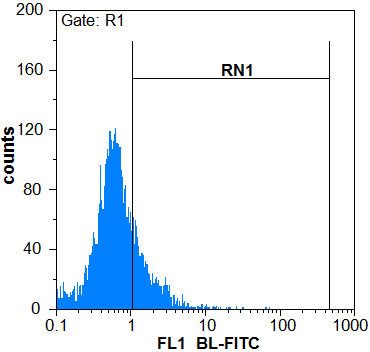

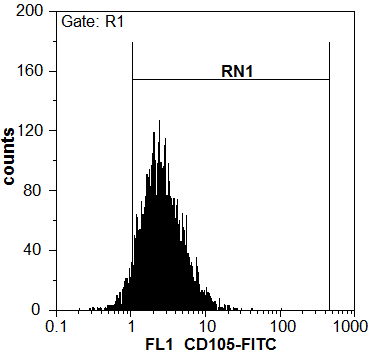

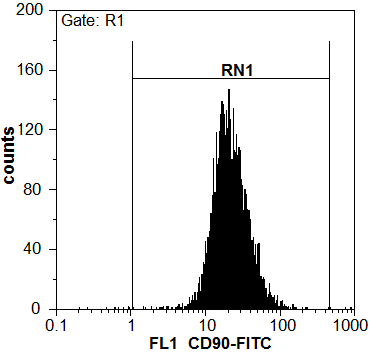

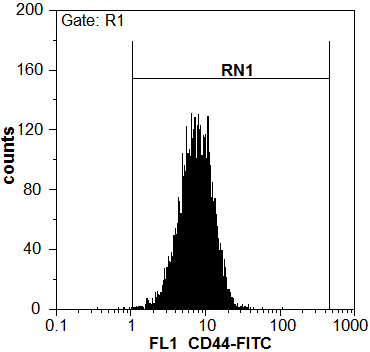

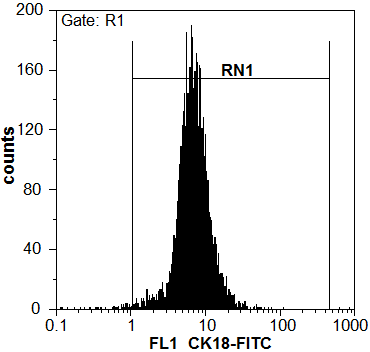

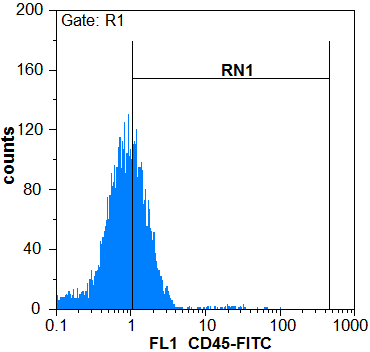

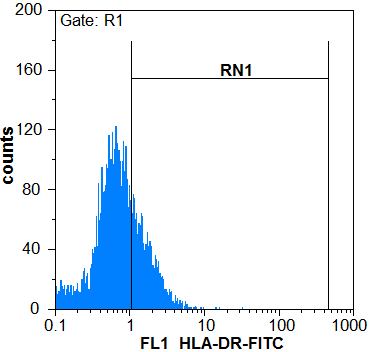

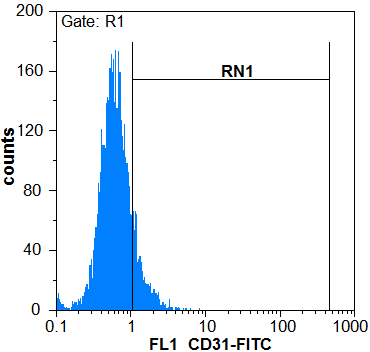


Additional file 1: Flow cytometry analysis of mesenchymal stem cells surface markers. The flow cytometry analysis shows expression of CD105, CD90, CD44, and CK18, and non-expression of CD 45, HLA-DR and CD31 on the hUCMSCs surface.
