# Supplementary material for: Pyroptosis of MCF7 Cells Induced by the Secreted Factors of hUCMSCs
Source: Stem Cells Int. 2018 Nov 11;2018:5912194. doi: 10.1155/2018/5912194 (PMC6252231; doi:10.1155/2018/5912194)
Supplement: Supplementary 2 — Additional file 2: confirm the phenotype using gradually increased ratio of hUCMSC-medium to fresh medium. Control: 100% fresh medium, treatment 1: 10% hUCMSC-medium + 90% fresh medium, treatment 2: 20% hUCMSC-medium + 80% fresh medium, treatment 3: 40% hUCMSC-medium + 60% fresh medium, treatment 4: 60% hUCMSC-medium + 40% fresh medium, treatment 5: 80% hUCMSC-medium + 20% fresh medium, treatment 6: 100% hUCMSC-medium. [file 5912194.f2.docx]

Day 1 Day 2 Day 3 Day 4 Day 5


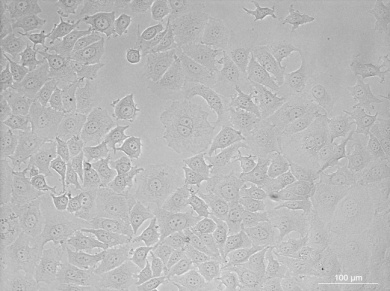

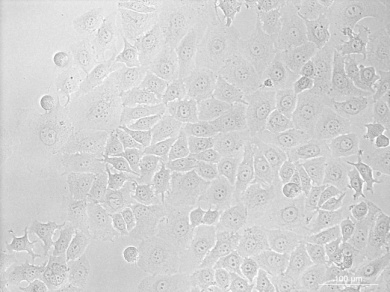

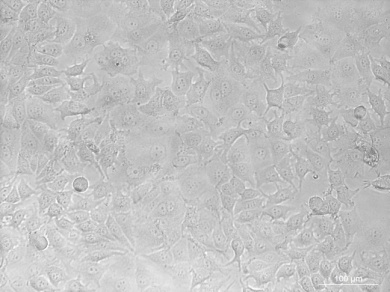

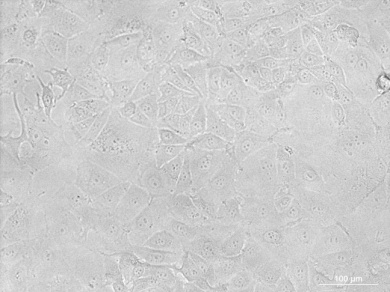

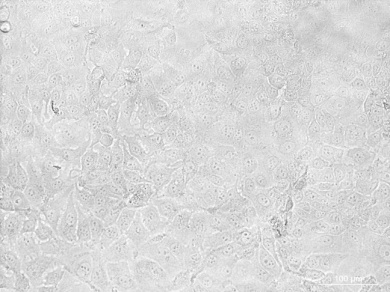


Control


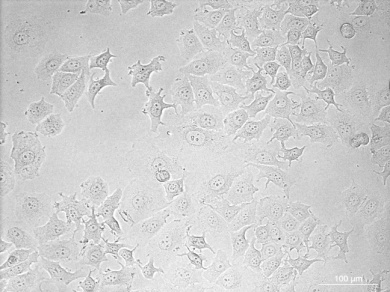

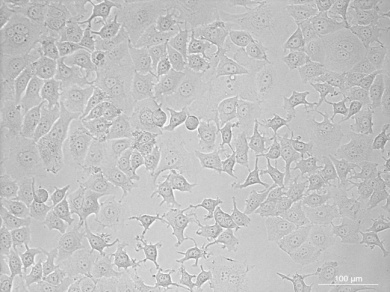

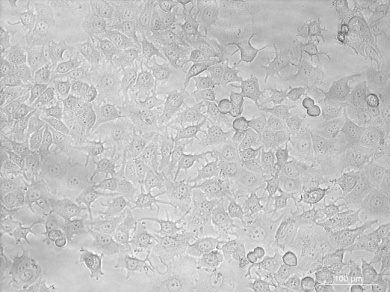

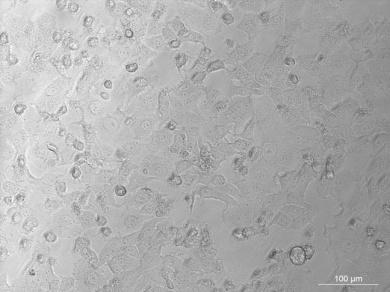

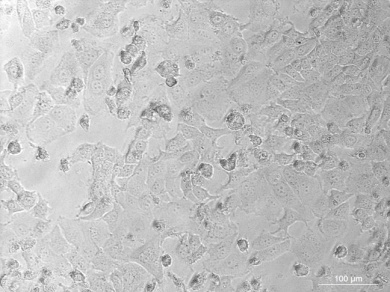


Treatment 1


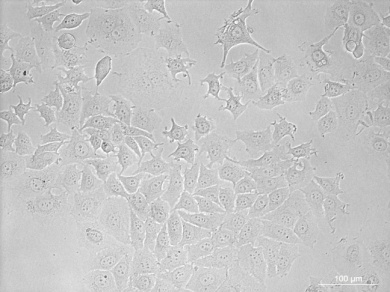

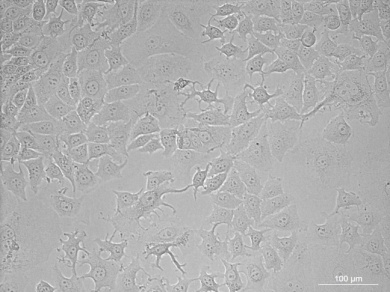

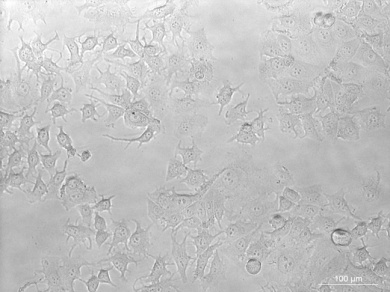

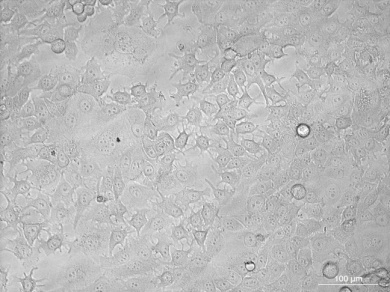

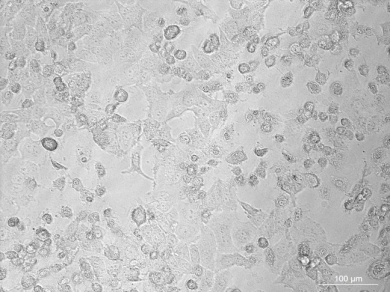


Treatment 2


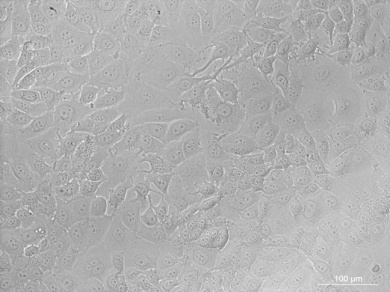

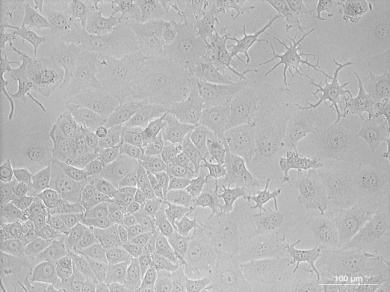

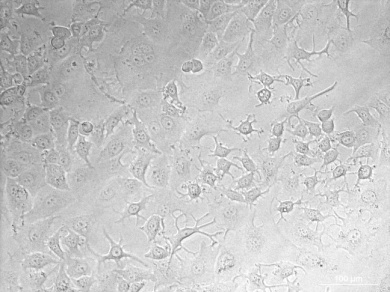

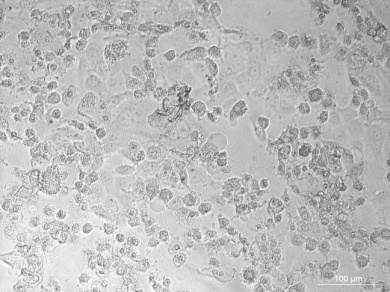

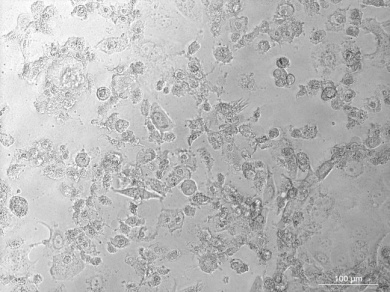


Treatment 3


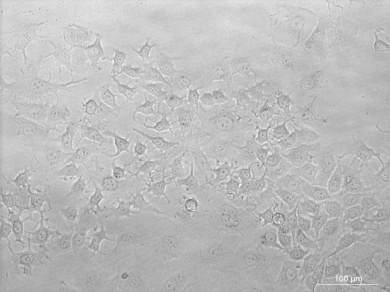

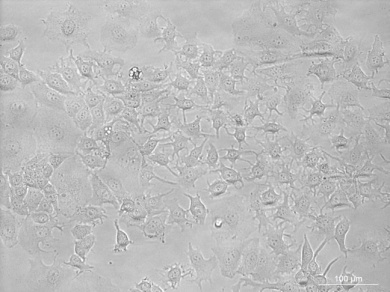

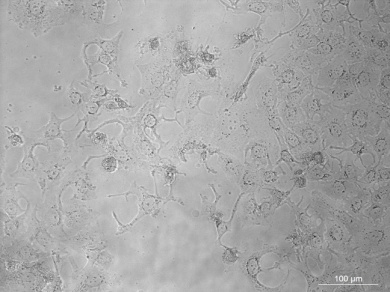

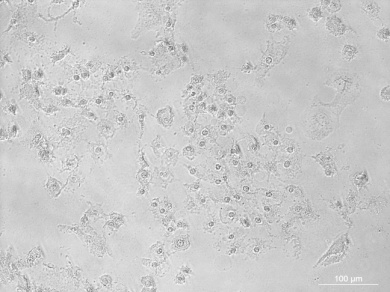

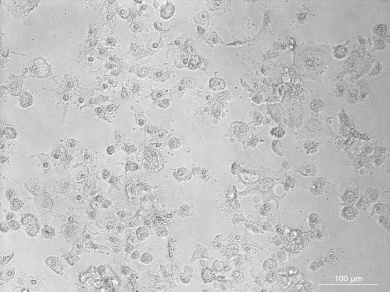


Treatment 4


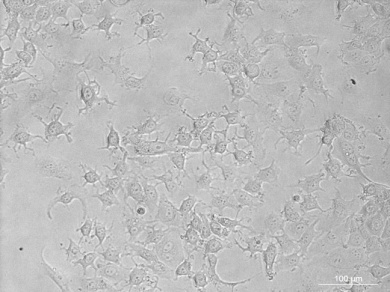

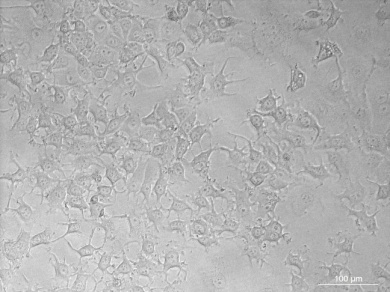

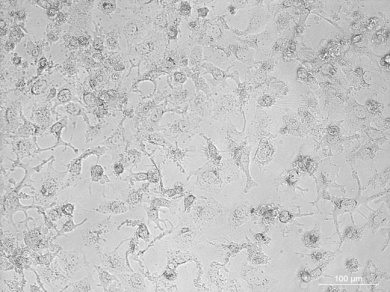

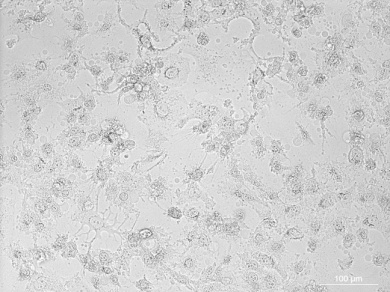

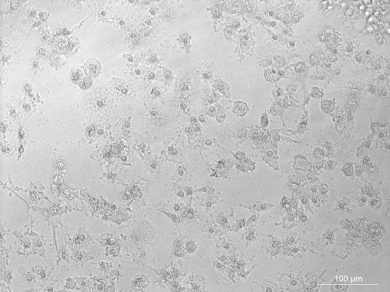


Treatment 5


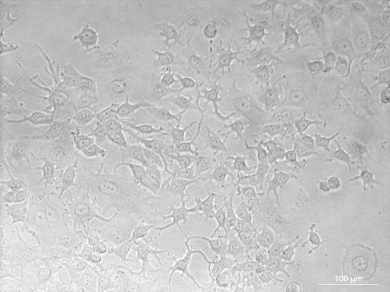

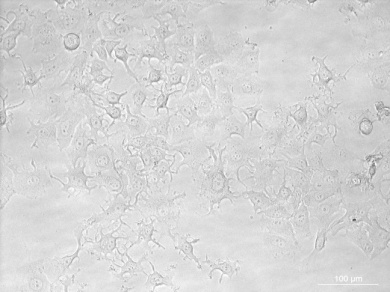

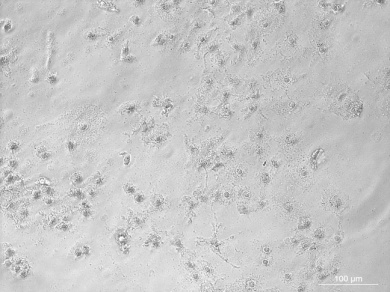

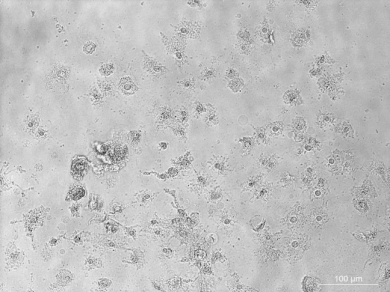

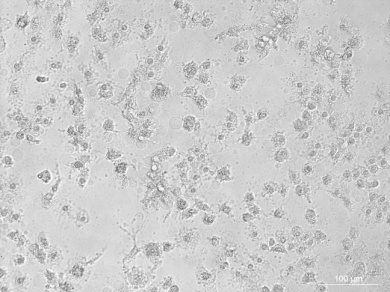


Treatment 6

Additional file 2: Confirm the phenotype using gradually increased ratio of hUCMSC-medium to fresh medium. Control: 100% fresh medium, Treatment 1: 10% hUCMSC-medium +90% fresh medium, Treatment 2: 20% hUCMSC-medium+80% fresh medium, Treatment 3: 40% hUCMSC-medium +60% fresh medium, Treatment 4: 60% hUCMSC-medium + 40% fresh medium, Treatment 5: 80% hUCMSC-medium +20% fresh medium, Treatment 6: 100% hUCMSC-medium .
