# Supplementary material for: Pyroptosis of MCF7 Cells Induced by the Secreted Factors of hUCMSCs
Source: Stem Cells Int. 2018 Nov 11;2018:5912194. doi: 10.1155/2018/5912194 (PMC6252231; doi:10.1155/2018/5912194)
Supplement: Supplementary 3 — Additional file 3: confirm the specificity of q-PCR primers using regular PCR. DNA marker: DL2000. [file 5912194.f3.docx]

Marker Bcl2 HIF1A NF-κB BIRC3


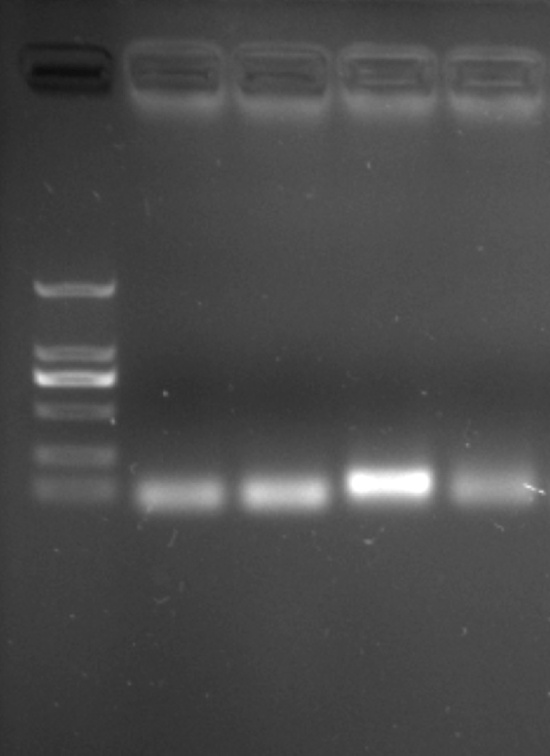


1000

750

500

250

100

bp

2000

Additional file 3 : Confirm the specificity of q-PCR primers using regular PCR. DNA marker: DL2000.
